# Evaluation of Enamel and Dentinal Microleakage in Class II Silorane-Based and Methacrylate-Based Resin Composite Restorations Using Specific and Nonspecific Adhesives

**Published:** 2018-07

**Authors:** Sayed Mostafa Mousavinasab, Maede Ghasemi, Mitra Yadollahi

**Affiliations:** 1 Professor, Dental Materials Research Center, Department of Operative Dentistry, School of Dentistry, Isfahan University of Medical Sciences, Isfahan, Iran; 2 Assistant Professor, Dental Materials Research Center, Department of Operative Dentistry, School of Dentistry, Isfahan University of Medical Sciences, Isfahan, Iran; 3 Assistant Professor, Department of Operative Dentistry, School of Dentistry, Shahrekord University of Medical Sciences, Shahrekord, Iran

**Keywords:** Silorane Composite Resin, Dental Leakage, Silorane System Adhesives, Polymerization

## Abstract

**Objectives:**

The aim of this study was to evaluate enamel and dentinal microleakage in Class II cavities restored with silorane- and methacrylate-based resin composites using specific and nonspecific adhesives.

**Materials and Methods:**

Thirty-six caries-free human premolars were used. Two Class II cavities were prepared on each tooth. The gingival floor was set at 1 mm above (on the mesial surface) and at 1 mm below (on the distal surface) the cementoenamel junction (CEJ). The samples were randomly divided into four groups, and the cavities were restored with a methacrylate-based composite (Filtek^™^ P60) and a silorane-based composite (Filtek^™^ P90) with specific and nonspecific adhesives. Microleakage was tested using a standardized dye penetration method. All samples were examined under a stereomicroscope, and microleakage scores were statistically analyzed using Kruskal-Wallis and Mann-Whitney-U tests. One sample from each group was examined under a scanning electron microscope (SEM) to determine the bonding area.

**Results:**

No significant difference was found between the groups in terms of the enamel microleakage (P=0.086). There was a significant difference between the groups with regard to dentinal microleakage (P=0.003). No significant reduction in microleakage was observed in groups restored with Filtek^™^ P90 composite using its specific adhesive compared to those restored with Filtek^™^ P60 composite using its specific adhesive (P=0.626).

**Conclusions:**

The results indicated that the application of methacrylate- and silorane-based composites with specific or nonspecific adhesives had no impact on enamel microleakage, but it affected dentinal microleakage, and specific adhesives showed less microleakage. It seems that a phosphate-methacrylate-based intermediate resin is required to bond dimethacrylate adhesive to silorane-based composites.

## INTRODUCTION

The resin matrix of a resin composite is an integral part of polymerization, which is mainly a di- or tri-ester of methacrylic acid. This class of materials has shown resistance in intraoral conditions since only methacrylates bond to different organic components such as aliphatic chains, polyesters, and aromatic rings [1].

In most commercial dental composites, bisphenol A-glycidyl methacrylate (Bis-GMA) is used as an organic matrix as its main advantages are a lower polymerization contraction than that of pure methacrylates as well as the high ability to cross-link [2]. One of the disadvantages of composite resins is the shrinkage resulting from the polymerization reaction, which leads to the accumulation of stress within the material and the tooth-restoration interface. Furthermore, if the stress level exceeds the bonding strength, a gap occurs in this area, which leads to leakage [3].

The attempts to improve the clinical efficiency and to remove internal stresses during the polymerization of methacrylate-based composites have led to the invention of novel polymerization systems such as silorane-based composites [4]. These composites are obtained from the reaction between oxirane and siloxane molecules. These composites have two advantages: firstly, the polymerization reaction is of a ring-opening type, in which the polymerization contraction is low due to the oxirane ring-opening compensatory mechanism [5], and secondly, the presence of siloxane leads to insolubility of the material in the presence of oral liquids, thereby increasing its hydrophobic properties [6]. Oxirane is a 3-membered cyclic ether monomer that is subjected to cationic polymerization; therefore, in addition to less polymerization contraction, cationic polymerization is not inhibited by oxygen in comparison to free radical polymerization [7]. Also, the presence of siloxane monomer in the composite, in addition to hydrophobicity, creates stability in this structure [8].

While methacrylate-based composites show a volume contraction equal to 2.3–3%, the silorane-based ones have been reported to show 0.9% volume contraction, which implies a lower level of stress to the cavity walls and reduced cusp bending [9].

Since the resin matrix in silorane-based composites is different than that of common methacrylate-based composites, a new adhesive called Silorane System Adhesive (SSA) has been designed and presented with this resin composite. This system is a two-step self-etch adhesive with similar characteristics of methacrylate-based adhesives in terms of the mechanism of bonding to the tooth. However, some changes have been made to this material to make it compatible with the hydrophobic silorane matrix. This primer has a pH of about 2.7, which generates a mild etching, a demineralized dental structure, and a strong and durable bonding [10,11].

Previous studies have shown higher marginal adaptation and less microleakage and cusp bending with silorane-based composites than with methacrylate-based ones [12]. Some studies have reported less microleakage and better marginal sealing for silorane-based than for methacrylate-based composites [13,14]. In contrast, Umer et al [15] showed that silorane-based composites did not have a better performance than methacrylate-based ones. Furthermore, Schmidt et al [16], in a randomized clinical trial, reported a better occlusal and proximal marginal adaptation with methacrylate-based composites.

They also found that reduction of polymerization contraction in the silorane group was not clinically significant [16].

Duarte et al [17] evaluated the nanoleakage and bond strength of a new low-shrinkage composite using different bonding methods in dentin. The results showed no bonding between Filtek LS composite and Adper single Bond Plus adhesive on the dentinal surface. Nonoleakage was also partially observed in all groups [17].

The current study was conducted to compare the microleakage of methacrylate-based and silorane-based composites used with their specific adhesives and to analyze their possible application using nonspecific adhesives. An attempt was also made to analyze the effect of using these composites with nonspecific adhesives on enamel and dentinal microleakage. The null hypothesis was that the use of specific or nonspecific adhesives has no effect on the enamel and dentinal microleakage of these two types of composite resin.

## MATERIALS AND METHODS

This study has been approved by the Ethics Committee for Research of Isfahan University of Medical Sciences (IRB No. 394611). A total of 36 newly-extracted premolars with no caries or fractures were collected. The teeth had been extracted due to orthodontic treatments during the past three months. To ensure adequate disinfection and to prevent cross-contamination, the teeth were cleaned and immersed in a large volume of freshly-prepared 0.5% chloramine-T solution.

Following this, they were placed in distilled water at 4°C for at least 2 hours [18]. Two standard Class II cavities (box only) were prepared on the mesial and distal surfaces of each tooth using coarse straight fissure diamond burs (ISO 806 314, Hager & Meisinger GmbH, Neuss, Germany). The bur was exchanged after every five preparations.

A buccolingual width of 2 mm and an axial depth of 1.5 mm were prepared in the cavities. The dimensions of the cavities were measured using a periodontal probe.

The gingival floor was set at 1 mm above the cementoenamel junction (CEJ) in the mesial cavity and at 1 mm below the CEJ in the distal cavity [15]. The samples were randomly divided into four groups, each with nine samples, and the cavities were restored as follows:

Group 1: Methacrylate-based composite resin (Filtek^™^ P60; 3M ESPE, St. Paul, MN, USA) with its specific methacrylate adhesive (control group).
Group 2: Silorane-based composite resin (Filtek^™^ P90; 3M ESPE, St. Paul, MN, USA) with its specific silorane adhesive (control group).
Group 3: Methacrylate-based composite resin with silorane adhesive.
Group 4: Silorane-based composite resin with methacrylate adhesive.

The methacrylate-based and silorane-based composite resins with their specific adhesives are presented in Table 1.

**Table 1:** Materials used in this study, and instructions for use

**Materials (batch number)**	**Manufacturer**	**Composition**	**Instructions for use**
Filtek^™^ P90 (4762I)	3M ESPE, St. Paul, MN, USA	**Silorane Resin** Initiating system: Camphorquinone, Iodonium salt, Electron donor Quartz filler Yttrium fluoride Stabilizers Pigments	The composite was used in three layers to restore the cavities, each layer being exposed for 20 seconds.
P90 System Adhesive (4763)	3M ESPE, St. Paul, MN, USA	**Self-Etch Primer:** Phosphorylated methacrylates Vitrebond^™^ copolymer BisGMA HEMA Water Ethanol Silane-treated silica filler Initiators Stabilizers **Bonding Agent:** Hydrophobic dimethacrylate Phosphorylated methacrylates TEGDMA Silane-treated silica filler Initiators Stabilizers	Apply the self-etch primer for 15 seconds, followed by gentle air dispersion and 10 seconds of light-curing. Then, apply the bonding agent followed by gentle air dispersion and 10 seconds of light-curing.
Filtek^™^ P60 (N661386)	3M ESPE, St. Paul, MN, USA	Bis-GMA Bis-EMA TEGDMA UDMA Nanofiller silica	The composite was used in three layers to restore the cavities, each layer being exposed for 20 seconds.
CLEARFIL^™^ SE BOND (6K0004)	Kuraray Noritake Dental, Tokyo, Japan	**Self-Etch Primer:** 10-MDP HEMA Water Photo Initiators **Bonding Agent:** 10-MDP Bis-GMA HEMA Hydrophilic dimethacrylate Microfiller	Apply the primer and leave for 20 seconds. Dry with mild airflow for 5 seconds. Apply the bonding agent and make a uniform bond film using a gentle airflow, and then, light-cure for 10 seconds.

HEMA=2-hydroxyethyl methacrylate, UDMA=Urethane dimethacrylate, Bis-GMA=Bisphenol A glycol dimethacrylate, TEGDMA=Triethylene glycol dimethacrylate, Bis-EMA=Ethoxylated bisphenol A glycol dimethacrylate, 10-MDP=10-methacryloxydecyl dihydrogen phosphate

After restoring of the prepared cavities, the samples were stored in distilled water for 24 hours, and then, all the samples were exposed to 1000 thermal cycles at 5°C to 55°C in a thermocycling machine (Delta Tpo2, Nemo, Mashhad, Iran) with a dwell time of 30 seconds. One sample from each group was selected for scanning electron microscopy (SEM; TESCAN, MIRA3, Kohoutovice, Czech Republic) to determine the bonding area, while the other samples were prepared for microleakage analysis under a trinocular zoom stereomicroscope (SMP-200, HP, USA) equipped with a digital camera (Moticam 480, SP10. 0224, Motic Instruments Inc., CA, USA).

The apices of the teeth were sealed with a layer of sticky wax, and all the surfaces, except for 1 mm around the tooth-restoration interface, were covered with two layers of nail polish. The teeth were then soaked in 0.5% alkaline fuchsine for 24 hours at 23°C. Next, the teeth were washed with distilled water, mounted in epoxy resin, and sectioned longitudinally in the mesiodistal plane using a low-speed diamond disc in a non-stop cutting machine (Dentarapid, Krupp Dental, 759DRZ, Hilzingen, Germany). The sectioned samples were analyzed under the stereomicroscope at 20× magnification, and the microleakage level was determined (Table 2) [19].

**Table 2:** Description of microleakage levels at gingival margins

0	No dye penetration
1	Dye penetration less than $\frac{1}{2}$ of the gingival wall
2	Dye penetration along the gingival wall
3	Dye penetration along the gingival wall and less than $\frac{1}{2}$ of the axial wall
4	Dye penetration along the gingival wall and the axial wall

**Fig. 1:**
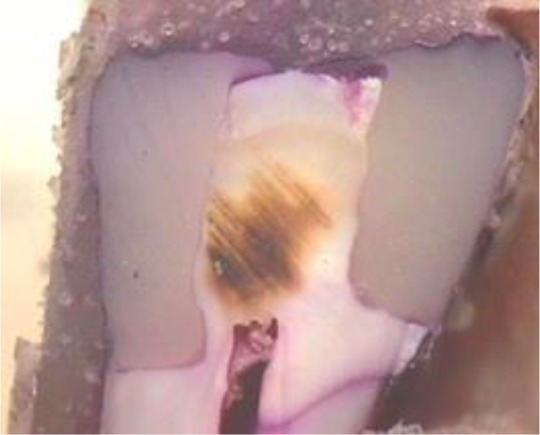
Degree 3 of microleakage under the CEJ (left side of the image), and degree 1 of microleakage above the CEJ (right side of the image)

Figure 1 shows the sections of a restored tooth and the microleakage level.

### Preparation of samples for SEM:

To analyze the samples by SEM, one sample was selected from each group (as mentioned in the previous section). To create a smooth surface on the enamel and dentin, all surfaces were polished using Sof-Lex^™^ (3M ESPE, St. Paul, MN, USA) paper disc series as rough, medium, soft, and very soft under running water. Afterwards, the samples were placed in an ultrasonic machine (TELSONIC AG, Bronschhofen, Switzerland) for 10 minutes to remove the particles produced during polishing. Then, to observe resin tags and their side branches at the enamel-dentin interface, the surfaces obtained from cutting the teeth were etched and demineralized by hydrochloric acid 6.00 Normal (6N HCl) for 30 seconds. Next, to remove the organic parts (collagen and protein), 2.5% sodium hypochlorite (NaOCl) was used for 10 minutes. Then, the samples were placed in a dry environment for 24 hours to completely dry. Between each stage, the samples were placed in the ultrasonic machine for 10 minutes. Finally, the specimens were gold sputtered, and the morphological evaluation of the resin-dentin interface was conducted using the SEM at various magnifications [20].

The data were analyzed in SPSS 21 software (SPSS Inc., Chicago, IL, USA) according to Wilcoxon, Kruskal-Wallis, and Mann-Whitney-U tests. The level of significance was primarily set at 0.05 in all tests; however, after Bonferroni correction, this level was reset at 0.008.

## RESULTS

The distribution of the enamel and dentinal microleakage scores in the four studied groups are presented in Table 3.

**Table 3:** Frequency of microleakage degrees in enamel and dentin in the studied groups

**Score**	**Groups**	**Degree of Microleakage N(%)**				

		**0**	**1**	**2**	**3**	**4**
**Enamel**	Filtek^™^ P60 + CLEARFIL^™^ SE BOND	3 (37.5)	5(62.5)	0(0)	0(0)	0(0)
	Filtek^™^ P90 + P90 System Adhesive	4(50)	3(37.5)	1(12.5)	0(0)	0(0)
	Filtek^™^ P60 + P90 System Adhesive	3(37.5)	1(12.5)	0(0)	2(25)	2(25)
	Filtek^™^ P90 + CLEARFIL^™^ SE BOND	0(0)	4(50)	4(50)	0(0)	0(0)
**Dentiun**	Filtek^™^ P60 + CLEARFIL^™^ SE BOND	4(50)	4(50)	0(0)	0(0)	0(0)
	Filtek^™^ P90 + P90 System Adhesive	5(62.5)	3(37.5)	0(0)	0(0)	0(0)
	Filtek^™^ P60 + P90 System Adhesive	0(0)	4(50)	0(0)	2(25)	2(25)
	Filtek^™^ P90 + CLEARFIL^™^ SE BOND	1(12.5)	4(50)	3(37.5)	0(0)	0(0)

Groups with different letters (ab) are significantly different in terms of the dentinal microleakage (P<0.008; Bonferroni method); N=Number

Wilcoxon test showed no significant difference between the enamel and dentinal microleakage in any of the samples (P=0.593). Furthermore, Spearman correlation coefficient revealed a significant correlation between enamel and dentinal microleakage (P<0.001, r=0.638).

Regarding the cavity restoration method, in comparing the four groups, Kruskal-Wallis test showed no significant difference between the groups in terms of the enamel microleakage (P=0.086). However, a significant difference was found between the groups with regard to dentinal microleakage (P=0.003).

The results of Mann-Whitney-U test on dentinal microleakage indicated less microleakage in group 1 (Filtek^™^ P90 composite with its specific adhesive) than in group 2 (Filtek^™^ P60 composite with its specific adhesive), but the difference was not statistically significant (P=0.626).

In general, the application of specific adhesives in dentin showed the minimum level of microleakage among the groups.

Group 3 (methacrylate-based composite/silorane adhesive) showed a significantly higher dentinal microleakage compared to group 1 (P=0.007) and group 2 (P=0.004).

**Fig. 2:**
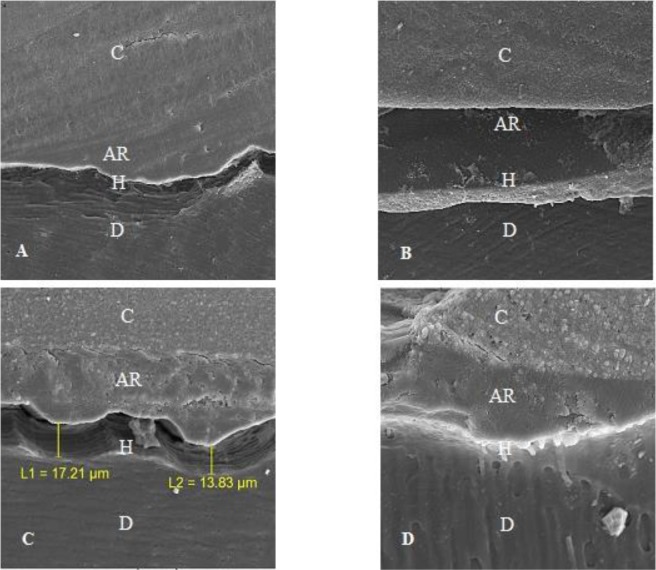
Scanning electron microscopy (SEM) images of the adhesive-composite interface in (A) group 1, (B) group 2, (C) Group 3, and (D) Group 4. C=Composite, AR=Adhesive Resin, H=Hybrid Layer, D=Dentin

Figure 2 shows SEM images of the adhesive-composite interface in the four groups. As compared to Figures 2a and 2b, discontinuous regions at the composite-adhesive interface are observed in Figure 2c. Resin tags, composite-adhesive bonding areas, and partially discontinuous regions are observed in Figure 2d.

## DISCUSSION

In this study, the microleakage of methacrylate-based and silorane-based composites used with their specific adhesives was evaluated, and their possible applications with non-specific adhesives were analyzed. An attempt was also made to analyze the effect of using different methods of applying the two different composite resins and bonding agents on microleakage in enamel and dentin. Based on the results, the null hypothesis on the effect of using different methods was accepted with regard to enamel microleakage; however, the hypothesis was rejected with regard to dentinal microleakage.

Composite restorations of posterior teeth have recently become popular because of their color matching with the teeth, thermal insulation, bonding to dental tissue, and being free from mercury [21]. Given the higher thermal expansion coefficient of restorative materials than that of dental tissue, frequent thermal stresses are generated at the tooth-restoration interface. These tensions may lead to the formation of cracks in the bonding area [21]. Therefore, since temperature changes can be an effective factor involved in the occurrence of microleakage, in the present study, a thermocycling was used to simulate temperature changes in the oral cavity.

When the enamel bond is exposed to stress by thermal cycles in the laboratory, self-etch adhesive systems are more destroyed compared to etch-and-rinse systems. [22] This reduction in the bonding strength, owing to thermal fatigue, can be indicative of the potential incidence of enamel microleakage during the application of self-etch systems. In a 10-year evaluation of self-etch primers (SEP), 39 cases out of 49 restorations showed marginal discoloration [22]. Use of phosphoric acid for enamel etching is preferable, and most studies have emphasized that applying a separate etching to enamel using phosphoric acid should be considered as one-stage bonding [23]. Hence, in the present study, enamel microleakage was not found to be significantly different than dentinal microleakage due to the absence of a separate etching stage.

In deep cavities with a high C-factor, the layering technique is the most appropriate method of composite placement [24]. Hence, this method was used in the present study for restoration of cavities. The need for a specific adhesive to have an acceptable bonding strength has limited the general application of silorane. However, Tezvergil-Mutluay et al [25] showed that it is possible to bond dimethacrylate composite resin to silorane using an intermediate phosphate methacrylate resin. In addition, it is possible to bond a silorane composite to a dimethacrylate-based adhesive using a phosphorylated methacrylate such as P90 System Adhesive (PSA) as an intermediate resin [17]. PSA is a methacrylate-based adhesive; therefore, it is compatible with common methacrylate-based composites [26]. According to the manufacturer’s instructions, Filtek^™^ P90 composite needs to be used with PSA in order to make an ideal restoration. However, despite the fact that Filtek^™^ P90 composite needs PSA, it is possible to use PSA with methacrylate-based composites owing to the common methacrylate base [27].

As indicated by Gao et al [27], PSA in combination with Clearfil AP-X (Kuraray) and Quixfil (Dentsply) methacrylate composites showed a better marginal adaptation than XP Bond Adhesive (XBA) and an equal marginal adaptation with CLEARFIL^™^ SE BOND. The results of the current study are in line with those of other similar studies [28,29].

In the current study, a significant difference was observed among the four studied groups in dentinal microleakage, and the application of silorane composite with its specific adhesive showed the minimum level of microleakage. However, no significant difference was found in the microleakage level between Filtek^™^ P60 composite/Clearfil^™^ SE Bond and Filtek^™^ P90 composite/P90 System Adhesive (groups 1 and 2).

The degree of microleakage was higher with Filtek^™^ P60 and Filtek^™^ P90 composites used with nonspecific adhesives. However, despite subjecting the samples to 1000 thermal cycles, no debonding or bonding failure occurred, which is indicative of the adaptability of these adhesives with their two nonspecific composites. Furthermore, despite the higher mean microleakage degree in these groups, samples with 0-degree microleakage were also observed, which indicate that silorane adhesive is methacrylate-based and compatible with methacrylate composites.

As shown in previous studies, an intermediate methacrylate-phosphate resin, similar to silorane bonding, is required with Filtek^™^ P90 composite when a methacrylate adhesive is used [17]. As Clearfil SE Bond is a methacrylate-based adhesive and contains 10-methacryloyloxydecyl dihydrogen phosphate (10-MDP) functional monomer (Table 1), the phosphate group of this functional monomer seems to be involved in the bonding of this adhesive to silorane composite resin, bringing about the adaptation of this adhesive with P90 composite.

In addition to the compatibility between methacrylate composite and silorane adhesive, it seems that the higher viscosity of the phosphate-methacrylate silorane resin, compared to dimethacrylate-based resins, acts as an elastic layer and, to some extent, compensates for the contraction stress generated at the interface, which is the result of polymerization of free radicals in methacrylate-based composite. Thus, the contraction stresses in methacrylate-based composites can partially be absorbed by this intermediate layer with a low modulus [27].

The SEM images of group 3 show the bonding area of silorane adhesive to methacrylate composite, although there is a discontinuity between these two layers in some areas. The bonding areas partly showed the bond between these two layers, which is due to their methacrylate structure.

In group 4, similar to group 3, bonding was made between methacrylate adhesive and silorane composite. Discontinuities were observed between these layers in some regions; however, they were less frequent compared to group 3. Continuous areas are indicative of the bonding of phosphate group adhesive to the oxirane ring of the composite.

Although the comparison of microleakage in groups 3 and 4 showed no significant difference, the microleakage of group 4 was rather lower. The SEM images also revealed fewer discontinuous areas in group 4 than in group 3. It can be inferred from these results that the adequacy of Clearfil^™^ SE Bond with silorane composite (Filtek^™^ P90) is better than that of P90 System Adhesive with methacrylate composite (Filtek^™^ P60).

In a study by Samimi et al [30], the effect of different bonding strategies on the micro-shear bond strength of a silorane-based composite resin to dentin was evaluated. Similar to the current paper, the groups consisted of silorane composite resin with and without its specific adhesive as well as methacrylate composite resin with its specific adhesive. Dissimilar to the present study, the use of methacrylate composite resin with silorane adhesive was not evaluated, and different bonding strategies were only compared in dentin as substrate. The results of their study in similar groups were in line with that of the present study, indicating no significant differences between the bonding groups (P=0.06).

Despite the improved characteristics of composites, factors such as chipping, discoloration, and fracture of composites are still of great concern, and dentists should decide to use either a substitute restoration or a repair [31]. Since removing all the composite causes an increase in the size of the cavity and changes the structure of the tooth, repair is often preferable to the replacement of composite [31]. When silorane composite needs repair, dentists cannot differentiate it from methacrylate composites owing to the similar appearance [31]. The findings of the current study can partially be used in case of repairing a silorane-based composite. When repairing a composite, if it is not ensured that the composite is methacrylate-based, using a bonding agent with a phosphate group (such as a bonding containing 10-MDP) and/or silorane bonding agents is preferable; if the old composite is silorane-based, it is more compatible with a silorane bonding, and also, the new methacrylate-based composite is quite compatible with a silorane bonding. Moreover, since silorane bonding agent is, in fact, a methacrylate-phosphate silorane resin [17], P90 adhesive can be used as a bridge between silorane and methacrylate composites.

The results of our study are also applicable when methacrylate composites and their specific silorane adhesives are not available or in cases where a combination of the two composites is used for aesthetic purposes.

Although the present study showed no significant difference between enamel and dentinal microleakage, future studies with larger sample sizes or using a different etching technique on enamel are recommended. In the present study, the compatibility of silorane composite resins with nonspecific adhesives was more attributable to the reaction of the phosphate adhesive group with this type of composite. Hence, further studies are suggested to apply methacrylate-based adhesives without a phosphate group (10-MDP) together with silorane adhesive in order to analyze their adaptability with silorane composites.

## CONCLUSION

The results of this study indicated that the application of methacrylate- and silorane-based composite resins with specific or nonspecific adhesives had no impact on enamel microleakage, but it affected Dentinal microleakage. Less microleakage was observed with specific adhesives.
